# Identification of Neoantigens in Cancer Cells as Targets for Immunotherapy

**DOI:** 10.3390/ijms23052594

**Published:** 2022-02-26

**Authors:** Masahiro Okada, Kanako Shimizu, Shin-ichiro Fujii

**Affiliations:** 1 Laboratory for Immunotherapy, RIKEN Center for Integrative Medical Sciences, 1-7-22, Suehiro-cho, Tsurumi-ku, Yokohama 230-0045, Japan; masahiro.okada@riken.jp (M.O.); kanako.shimizu@riken.jp (K.S.); 2 Program for Drug Discovery and Medical Technology Platforms, RIKEN, 1-7-22, Suehiro-cho, Tsurumi-ku, Yokohama 230-0045, Japan

**Keywords:** neoantigens, ICB, vaccines

## Abstract

The clinical benefits of immune checkpoint blockage (ICB) therapy have been widely reported. In patients with cancer, researchers have demonstrated the clinical potential of antitumor cytotoxic T cells that can be reinvigorated or enhanced by ICB. Compared to self-antigens, neoantigens derived from tumor somatic mutations are believed to be ideal immune targets in tumors. Candidate tumor neoantigens can be identified through immunogenomic or immunopeptidomic approaches. Identification of neoantigens has revealed several points of the clinical relevance. For instance, tumor mutation burden (TMB) may be an indicator of immunotherapy. In various cancers, mutation rates accompanying neoantigen loads may be indicative of immunotherapy. Furthermore, mismatch repair-deficient tumors can be eradicated by T cells in ICB treatment. Hence, immunotherapies using vaccines or adoptive T-cell transfer targeting neoantigens are potential innovative strategies. However, significant efforts are required to identify the optimal epitopes. In this review, we summarize the recent progress in the identification of neoantigens and discussed preclinical and clinical studies based on neoantigens. We also discuss the issues remaining to be addressed before clinical applications of these new therapeutic strategies can be materialized.

## 1. Introduction

### Clinical Significance of Tumor Neoantigens

**Figure 1:**
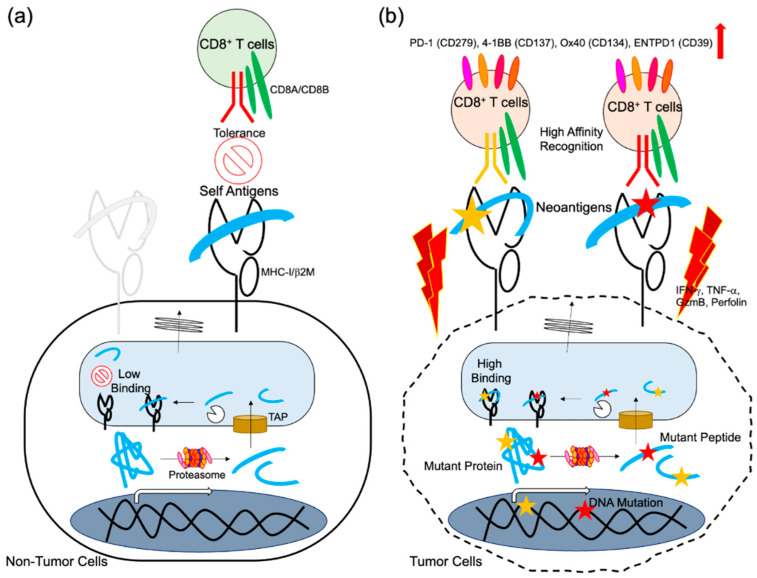
Neoantigen presentation and T cell responses. Cellular proteins are degraded by the Ub-proteasome. Some peptide products are transported and further processed in the ER, then loaded onto MHC-I, and presented on the cell surface. (**a**) Autologous T cells cannot recognize self-antigens. In contrast, (**b**) mutant proteins resulting from tumor somatic mutations yield mutant peptides, which facilitate MHC-I interaction or TCR recognition depending on the mutant position. By responding to the neoantigen, T cells proliferate and show activated phenotypes with tumoricidal capacity.

Tumor-specific somatic mutation-derived antigens (neoantigens) are newly synthesized in tumors and recognized as non-self. By targeting neoantigens, the T cells can attack and kill tumors [1,2,3]. Clinical studies have reported successful therapeutic outcomes of immune checkpoint blockage (ICB) for tumor treatment [4,5]. Various immune cells show anti-tumor immune responses in the tumor microenvironment and lymph nodes, immune cells with direct tumor killing activity are essential for the eradication and suppression of proliferating tumor cells. In particular, CD8^+^ T lymphocytes exhibit tumor selectivity and high cytotoxic activity. CD8^+^ T cells become dysfunctional following chronic antigen exposure, and ICB treatment reinvigorates tumor-specific T cells by inhibiting signaling-mediated suppression. CD8^+^ T cells often recognize over-expressed self-antigens in tumors, such as cancer testis antigens, exogenous onco-virus antigens, and tumor neoantigens [6]. Since CD8^+^ T cells are educated to have central tolerance, viral-associated antigens or neoantigens are expected to be the ideal targets (Figure 1).

A large number of tumor DNA mutations potentially yield mutated peptide sequences. Tumor mutation burden (TMB) alters neoantigen load and immunogenicity; hence, tumors exposed to mutagens, such as skin and lung tumors (UV and cigarette, respectively), are proactively treated by ICB [7,8,9]. Additionally, ICB therapy reinvigorates neoantigen-specific T-cells, supporting their importance in killing tumor cells [10]. Indeed, a higher TMB is associated with clinical responses to ICB [8,11]. Mismatch repair-deficient tumors, which likely accumulate mutations during cell division, are susceptible to ICB treatment [12,13]. Tumor inhibition by ICB is partly accounted for by indel mutations and missense mutations. Therefore, microsatellite instability (MSI) may be a plausible biomarker for ICB therapy [14]. In contrast, patients respond variably to ICB treatment, regardless of the mutation burden. Since the responses to neoantigens vary in patients, the tumor type and mutation burden may not be the only factors influencing these responses [15]. In fact, a high mutation burden is reportedly a risk factor for multiple myeloma [16]. Given the tumor diversity, anti-tumor immune responses mediated by neoantigens need to be thoroughly investigated. In this review, we focused on the current progress made in neoantigen identification in mice and human-based studies, and the complexity of immune responses. We also discuss potential neoantigen targeting strategies.

## 2. Neoantigen Identification Methodology Development

**Figure 2:**
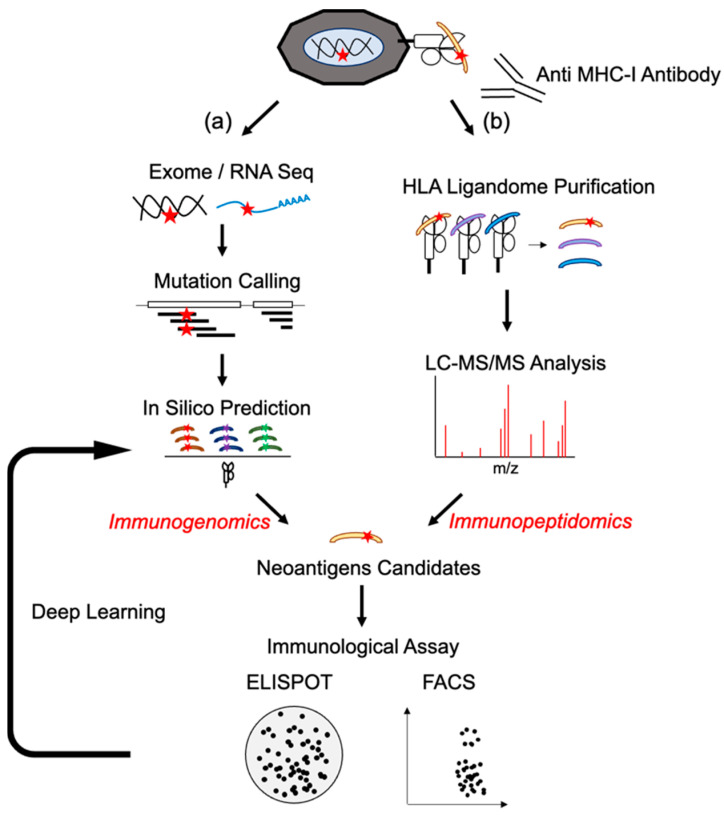
Neoantigen identification by immunogenomic or immunopeptidomic method. Tumor biopsy samples are analyzed by immunogenomic or immunopeptidomic method. (**a**) In immunogenomic method, tumor and matched germinal tissues are subjected to exome and tumor RNA-seq to detect somatic mutations in expressed genes. Overlapping missense or indel mutation peptide sequences are analyzed to predict affinity to each MHC-I allele. (**b**) In the immunopeptidomic method, tumor tissues are lysed, and peptide/MHC-I complexes are purified by immunoprecipitation using anti-MHC-I antibodies. Binding peptides are eluted and separated by size. Then, mass spectrometry is performed to determine molecular weight and identifying corresponding mutated peptides. Using candidate neoantigen peptides, T cell responses are investigated by evaluating cytokine production, activation marker expression, and tetramer staining. Validated neoantigen peptide data are subjected to machine learning analysis.

There are two major techniques to identify neoantigen epitopes, one based on genomic sequences and the other based on MHC-loaded peptides (Figure 2). The current status of both is described in the following sections.

### 2.1. Next-Generation Sequences and Epitope Prediction as Immunogenomics Approach

Researchers are seeking tumor-specific and mutation-derived antigens. Researchers have screened patient-derived tumor infiltrating lymphocytes (TIL) reactivity using a library constructed from tumor samples as well as cDNA codes simultaneously derived from tumor-specific mutations. p16INK4a-resistant CDK4 variant or mutated β-catenin are successful examples of neoantigens, and studies elucidating these were the foundation of recent progress [17,18]. Evaluating the genetic differences between tumor tissue and normal tissue using next-generation sequencing (NGS) significantly accelerated identification of neoantigens. Generally, researchers focus on the whole-exome and whole-transcriptome sequences. The sequence data are mapped onto the reference genome. The tumor and germline datasets are then parsed using somatic mutation callers. The mutant allelic frequency can be calculated using the mutation caller, and the mutation sites are determined. Transcriptome sequences also validate the expression of mutant genes. These mutated nucleotide-coding peptides are then further analyzed to identify neoantigens.

### 2.2. HLA Typing for Neoantigen Detection

Similar to other antigens, neoantigens are typically presented by major histocompatibility complex class I (MHC-I) for CD8^+^ T cells and MHC-II for CD4^+^ T cells in a cell-restricted manner. In humans, the diversity of human leukocyte antigens (HLA)-I (HLA-A, -B, and -C) and HLA-II (HLA-DR, HLA-DQ, and HLA-DP) exceeds 10,000 alleles [19]. Hence, HLA typing is necessary before the prediction of potential neoantigens. Although the input sequence data (exome vs. transcriptome) required for accurate HLA typing depends on the operating system, many analytical tools have been developed. Accurate HLA typing can be achieved using programs such as seq2HLA [20], Optitype [21], HLAProfiler [22] and arcasHLA [23]. In mice, MHC-I (H-2K, -2D, and -2 L) and MHC-II (I-A, I-E) differ among various strains.

### 2.3. In Silico Prediction of Neoantigens

Antigen presentation is regulated by multiple processes. Most proteins are degraded in the proteasome and processed into short length (8-11 AA) peptides [24]. In humans, some of these peptides are transported into the endoplasmic reticulum by a transporter associated with antigen processing (TAP) system and loaded on HLA-I. These HLA-I-peptide complexes are then expressed on the cell surface and recognized by cytotoxic cells (Figure 1). Additionally, the number of each human HLA class I allele exceeds 1000, and their combination yields polymorphic diversity. There are many computational prediction tools for antigen processing (NetChop [25]), peptide transport (NetCTL, NetCTLpan [26]), and peptide binding to MHC-I (NetMHC, NetMHCpan [27,28], MHCflurry [29], and SMMPMBEC [30]). Nevertheless, it is difficult to choose the optimal computation algorithm to predict the peptide for loading onto MHC-I, since there are multiple parameters for determining an MHC-I-peptide complex. Despite their optimization, a limited number of predicted epitopes are presented on cell surfaces [31]. In addition, unbiased neoantigen screening can successfully identify clinically relevant epitopes that cannot be predicted by conventional algorithms [32]. This implies that optimizing the binding affinity alone does not reflect the actual cellular processing and CD8^+^ T cell responses. Currently, deep learning by importing HLA-ligandome data is being applied to optimize the prediction capacity [33,34,35,36]. Continuous improvement in the precise prediction of neoantigens will allow for the versatile application of neoantigen-based vaccines. In fact, current clinical trials have relied on in silico prediction algorithms for selecting neoantigen candidates.

### 2.4. Mass Spectrometry Analysis (Immunopeptidomics)

Another approach that has been utilized involves mass spectrometry (MS) analysis of the MHC-I ligandome. In contrast to prediction algorithms using exome and transcriptome, MHC-I-loaded peptides that are naturally processed in the cells can be directly identified. For this, MHC-I ligandomes are immunoprecipitated, and the eluted peptides are analyzed by LC-MS/MS. This detailed method has been extensively reviewed [37,38]. Researchers have found that, compared with many predicted neoantigens, a lower number of neoantigens were actually present on MHC-I [39,40]. Moreover, it is noteworthy that neoepitopes identified by MS explicitly mediate tumor rejection despite the weak binding affinity, as predicted [41]. The difficulty in preparing samples from tumor tissues depends not only on tumor volume, but also on efficient immunoprecipitation and elution. The mass peak analysis strategy is decisive for the identification of low affinity but abundant peptides by combining NGS and in silico prediction in which neoepitopes can be directly uncovered [42,43]. In general, MS analysis usually requires many more cells than in silico prediction [44]. However, one report proposed that neoepitopes could be identified even in small samples of human melanoma tissues, and some mutated ligands in the patient’s tumor were immunogenic [45]. Furthermore, in addition to missense mutation neoantigens, MS analysis can identify immunostimulatory antigens derived from noncoding regions that cannot be identified by classical exome sequencing [46]. Despite several limitations, such as the threshold for detection and lower throughput than in silico prediction, we can identify a few, but real neoepitopes in tumor patients with MS.

## 3. Neoantigen-Specific T Cell Responses

### 3.1. Reactivity of Neoantigen-Specific T Cells after Vaccines

After neoantigen candidates are identified by in silico prediction or MS analysis, the next important step is to determine if the epitope can be directly recognized by T cells. Generally, in mouse experiments, synthesized peptides or coding RNAs are used to immunize and T cell reactivity against cognate peptides is monitored by IFN-γ production [39,47,48,49,50]. The tumoricidal potential against widely examined tumor cell lines can be evaluated regardless of the adjuvants used. Moreover, even though these epitopes are predicted to have high MHC-I affinity, synthetic long peptides or long neoepitope-coding RNA vaccines can elicit MHC-II-restricted CD4^+^ T cell responses. With regard to the role of these neo-Ag specific CD4^+^ T cells in anti-tumor immunity, it has not been explained completely. Since most tumors lack MHC-II, tumor-infiltrating antigen-presenting cells (APCs) should express tumor-derived antigen including neo Ags on MHC-II. Therefore, CD4^+^ T cells may help CD8^+^ CTL via APC activation by CD40 ligand as well as IL-2 and IL-21 secretion [51,52,53]. Furthermore, several reports support the direct tumoricidal activity of CD4^+^ T cells against certain MHC-II expressing tumors including neoantigens [54,55,56]. In fact, mutant MHC-II neoepitope vaccine elicited anti-tumor response in CD4^+^ T cell-dependent manner [57]. DNA delivery vaccines effectively induce CD8^+^ T cell responses [58]. Despite T cell activation, it is unclear if neoantigen vaccines can sufficiently lead to tumor rejection [59]. In other experiments, a discrepancy between T cell responses and tumoricidal activity by vaccines using neoantigens has been reported [32,48].

The clinical trials of neoantigen-based vaccines are listed in Table 1. Neoantigen-pulsed dendritic cell vaccines promoted neoantigen-specific T cell frequency in patients with advanced melanoma [60]. Subsequently, a clinical study of a vaccine against melanoma showed that pooled neoantigen candidates immunized with poly ICLC achieved remarkable clinical responses by inducing antigen-specific polyfunctional T cells against tumors [61]. A clinical study using RNA-based vaccines also showed sustained progression-free survival in some patients with melanoma whose neoepitope-specific T cells killed the autologous tumor [62]. In gliomas with typically lower TMB, vaccination using neoantigens generated objective responses, the increase in tumor infiltrating lymphocytes (TILs), or elicitation of epitope-specific T cell responses against peptides [63,64,65]. Recently, there was a successful study on neoantigen vaccines in combination with anti PD-1 antibody treatment. The study showed that neoantigen vaccines elicited neoantigen T cell responses against new ones that had not been included in the antigen of the original vaccine [66]. Not only personalized vaccines, but also off-the-shelf vaccines using neoantigens designed from hot-spot mutations or frameshift mutations have been shown to be safe and feasible [67,68]. Of note, CD4^+^ T cells are frequently activated by neoantigen vaccines in certain patients as well as in mice preclinical studies. Despite the number of successful examples, it is a fact that not all patients have achieved clinical benefits. The vaccines certainly induce neoantigen-specific T cell responses, but the clinical benefits are limited in the number of patients with cancer. This implies that an infallible selection strategy for neoantigen candidates is required for precision vaccination in future clinics [69,70]. In addition to epitope immunogenicity, patients’ T cells, primed by vaccines, need to be evaluated to determine if they can respond to naturally processed tumor neoantigens before using the vaccines.

### 3.2. Existence of Neoantigen-Specific T Cells in Cancer Patients without Vaccines Treatment

**Figure 3:**
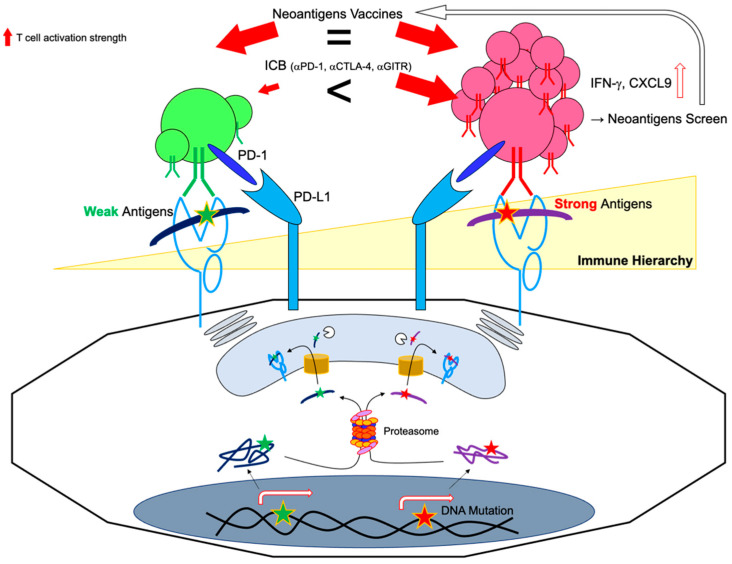
Neoantigen hierarchy. Most tumors present dominant and subdominant antigens on the surface. (i) PD-L1 on tumors suppresses neoantigen-reactive T cells. (ii) Immune checkpoint blockage preferentially reinvigorates dominant antigens. The subdominant neoantigens are also discovered by a highly sensitive neoantigen screen. Subdominant neoantigen-specific T cells can be activated by vaccines and dominant ones and can attack tumors.

Efficient sampling of tumor-reactive T cells from patients has revealed clinically relevant neoantigen responses. However, it has been reported that low or rare TILs can recognize autologous tumors in ovarian and colorectal cancers [74]. Many solutions have been proposed to overcome the limitations of low availability and low reactivity. Researchers have substituted healthy donors for wide range and robust identification, since there is a risk of underestimation in the use of patient-derived T cells [75]. Despite the limited number of tumor-reactive T cells in patients, it is noteworthy that ICB treatment strongly increased tumor-specific T cells, including neoantigens, in humans and mice [10,76]. Moreover, neoantigen-specific T cells have been identified in TILs from ICB-sensitive tumors compared to ICB-insensitive tumors in a mouse model [10,77]. Additionally, pre-existing neoantigen-specific T cells were reported as a decisive factor for successful immunotherapy outcomes [78]. An enhanced neoantigen immune response in the presence of ICB treatment was more strongly linked to CXCR3 ligands (CXCL9 and CXCL10) than IFN-γ, which enabled sensitive neoantigen detection in a mouse model [79]. To augment immune responses against tumor antigens by ICB treatment, we also investigated robust T cell expansion in PD-L1-deleted MC38 tumors, but not parental tumors. Utilizing expanded neoantigen-specific T cells in PD-L1-deficient tumor-bearing mice led to the identification of neoantigens that sufficiently attenuated tumor growth following dendritic cell-based vaccines (Figure 3) [80]. Several cell surface expression molecules, such as CD137, PD-1, CD39, and CD134, are used as potential activation markers to detect neoantigen-specific T cells (Figure 1) [67,81,82,83]. Analysis of the responses against mutation sequences, peptides, and tandem minigenes can help to identify clinically relevant neoantigens for precision medicine [67,84,85]. Tetramer-based detection and sorting of expanded neoantigen-specific T cells is feasible in both patients and healthy donors [86,87]. With respect to MHC-II neoantigens, CD4^+^ regulatory T cells (Tregs) in the tumor showed the tumor reactivity, especially for neoantigens. The repertoires of the Treg cells imply the potential target [88]. When patients respond to identified neoantigens, the anti-tumor immune responses are strengthened by these peptide vaccinations. In other cases, neoantigen-specific TCR-T adoptive transfer is expected to be an effective treatment.

## 4. Neoantigen Candidates as Shared Antigens

Most of the neoantigens are believed to be derived from passenger gene mutations. However, recent progress in human tumor studies has revealed that neoantigens derived from driver gene mutations could generate common and shared neoantigens in certain cases. *IDH1* R132H yields aberrant oncometabolite and induces gliomas, as observed in CD4^+^ T cells in patients and humanized mice carrying HLA-DRB1*01:01 [89]. This mutation-targeting peptide vaccine could elicit intratumoral inflammation in most patients harboring multiple HLA alleles [73]. Immunoglobulin-variable regions of lymphoma cells presented on HLA-DR*04:01 are recognized by cytotoxic CD4^+^ T cells [90]. H3.3 K27M mutation, which results in aberrant gene expression, is the cause of most diffuse intrinsic pontine glioma, and acts as the target of HLA-A2 restricted CD8^+^ T cells [91]. Frameshift mutant NPM1, which is frequently observed in acute myeloid leukemia, binds to HLA-A*02:01 [92]. Therefore, the TCR from the responding T cells was cloned. Moreover, TP53, a well-known mutated gene in many cancer types, was expressed on HLA-A*02:01 (R175H) and HLA-A*68:01 (R248W) (MHC-I) and HLA-DRB1*13:01 (R175H) and HLA-DRB3*02:02 (Y220C) and HLA-DPB*02:01 (R248W) (MHC-II) [93,94]. TCR against the mutated position of TP53 R175H has already been cloned and validated to recognize many kinds of tumors containing this same mutation [95]. Other famous driver mutations, KRAS G12D and G12V, were recognized by CD8^+^ and CD4^+^ T cells, respectively, in the specific alleles [96,97,98]. In addition, the other driver mutant PIK3CA and c-Kit are immunogenic in healthy donors [99]. Driver mutations are necessary to maintain tumor cell characteristics; therefore, more aggressive metastatic pancreatic cancers can harbor uniform gene mutations, thus supporting the shared neoantigens as strong therapeutic targets [100]. Missense and indel mutation-derived neoantigens are not limited, but fusion gene products, typically neighboring joint sequences, have recently been identified as tumor neoantigens even in tumors with low mutation burden tumors [101,102]. Fusion gene products are thought to be frequently involved in tumorigenesis [103]. Hence, fusion genes have become important in the novel neoantigen landscape for immunotherapy as well as driver mutation loci. These studies strongly suggest that NGS mapping should be performed over classical systems solely focusing on the exome to identify neoantigens. Beyond personalized medicine, shared neoantigens can become the primary choice for vaccine targets and neoantigen-specific TCR therapy.

## 5. Neoantigen Responsiveness and Clonality

While considering the usage of neoantigens for developing vaccines, physicians need to carefully consider immunodominance and tumor heterogeneity.

### 5.1. Immunodominant vs. Subdominant Neoantigens

**Figure 4:**
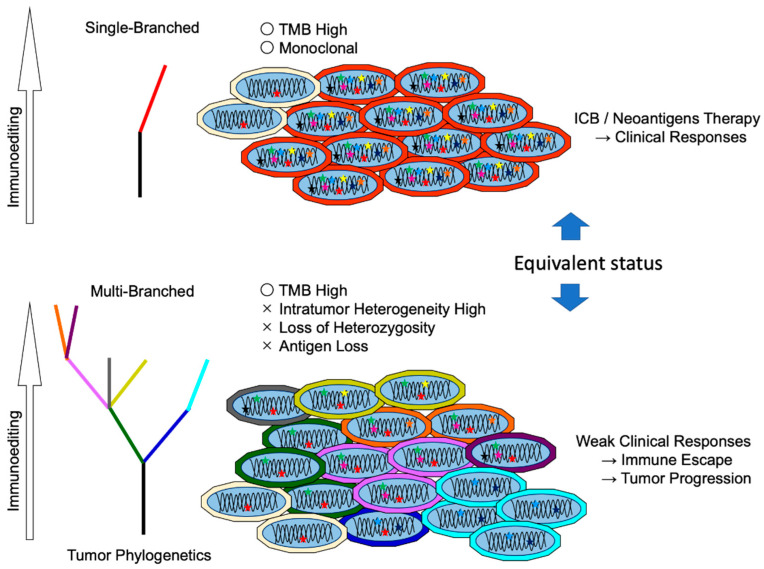
Relation between neoantigen clonality and intratumor heterogeneity. Immunoediting shapes tumor evolution. Immune cells eliminate neoantigens expressing tumor cells at early stages. (**a**) High mutation burden with monoclonal tumors is responsive to immunotherapy. Whereas (**b**) in progressed tumors, gain or loss mutations in tumor cells evade immune pressure. Beyond the equilibrium point, tumors aggressively proliferate, and heterogenous cancerous cells compose the tumor tissue. This ostensible high mutation burden with multiclonal tumors is resistant to immunotherapy.

A HLA-A2 restricted Matrix Protein epitope (M1 58-66) of influenza A induces robust T cell responses, whereas the other epitopes elicit weak responses.. The stability or abundance of peptide-HLA complexes, and the frequency or avidity of T cells in recognizing them presumably determine epitope hierarchy [104]. Since T cells responding to immunodominant antigen are spontaneously activated, they can kill target tumor cells. In contrast, subdominant epitopes often hinder reactivity at low levels by immunodominant epitopes, which are difficult to detect. Even if host immunity is sufficient, after evading immune defense against immunodominant epitopes, the subsequent immunity will become weaker than the previous one. Hence, subdominant epitopes are much less suited for tumor eradication in the absence of any treatments, triggering immune-escaped tumor progression. One report showed that subdominant T cell responses yielded incomplete differentiation, skewing to Tc17, and these kinds of T cell activation were evoked by direct vaccinations but not by ICB treatment, indicating the complexity of neoantigen targeting strategy (Figure 3) [105]. Although it most likely depends on individual cases, if tumors are stably composed of single clones, complete rejection will be achieved by immunodominant epitopes. However, if they multiply or evolve due to survival, complete rejection becomes difficult. Indeed, the loss of neoantigens is attributed to a reduction in RNA expression, and loss of mutant alleles is observed over the long term in some melanoma patients (Figure 4) [106].

### 5.2. Difference between Clonal Neoantigen and Subclonal Neoantigens

Multi-region analysis of high-grade serous ovarian cancer indicates that immune-selected tumors can evolve in patients with higher TIL density at the tumor interface [107]. Whereas tumor expressing subclonal neoantigens are likely to be killed, the clonal neoantigens in the remaining and proliferated tumor cells are less immunogenic, which leads to aggressive metastasis [108]. This was confirmed by analysis of the early stages of the tumor. In the early stage of non-small cell lung cancer (NSCLC), subclonal neoantigens were retained by a low number of TILs but were eliminated by a high number of TILs. Neoantigens identified in untreated early-stage NSCLC were also less overlapped in progressed tumors from TCGA data. Hence, the low number of TILs at the progressed stages was the result of evasion of the immune response [109]. Accordingly, immunoreactivity is spatially heterogeneous in biopsies from multiple loci of NSCLC, suggesting that analysis of multiple tumor lesions is needed for comprehensive prediction of neoantigen-based therapy [110]. In the experimental model, intratumor heterogeneity also reduced immune responses, which indicates the tumor neoantigen burden in the clonality determines the ICB responses. Hence, an ostensible higher mutation burden due to subclonality is implausible in predicting ICB outcomes (Figure 4) [111]. Recently, large-scale meta-analyses of ICB cohort studies clearly demonstrated that clonal TMB was the best predictor of ICB response, followed by total TMB, nonsense-mediated decay escape TMB, indel TMB, and subclonal TMB [112]. Therefore, despite the risk of weakening immunodominant TCRs, targeting multi-neoantigens appears to be cogent for therapeutic use.

Several mechanisms are involved in neoantigen loss. Allelic loss by mutagenesis, chromosome abnormality, and copy number loss by transcriptionally or epigenetically have been identified. A critical factor for immune evasion is the loss of heterozygosity (LOH) of HLA caused by defects in antigen presentation machinery or direct mutation in the HLA complex [113]. The HLA-I genotype determines the ICB responses. Maximal heterozygosity (HLA-A, B, and C) actually improves overall survival after ICB, but also results in some HLA type loss, leading to poor responses [114]. Therefore, immunoediting of various HLA polymorphisms in patients is a key factor for predicting anti-tumor immune responses.

## 6. Perspective

Tumor neoantigens are ideal targets for immunotherapy and are being actively pursued for designing cancer therapeutic strategies. Many clinical trials (phase I/II) based on neoantigens are currently ongoing and recruiting [115,116,117]. For a basic understanding of tumor immunobiology, identification of endogenous T cells directed against neoantigens at each tumor stage and each resected area at multiple loci is essential. Studies on human patients have demonstrated the complexity of tumor development under immunoediting. Additionally, clinically progressive tumors may have evolved during immunoediting. Therefore, it appears that patient-derived T cells have lost their potential to target tumors. In an experimental mouse model, most of the studies examined neoantigens in established tumor cell lines and did not follow naturally developing tumors. The analysis of neoantigen-specific T cell destiny in mice with spontaneous tumors is necessary for further comprehension. Moreover, as detected in shared neoantigens, the driver mutation became the target in some HLA-matched patients. This suggests the possible use of neoantigen vaccines for tumor protection before tumor appearance, similar to vaccines against oncoviruses, human papillomavirus (HPV), and hepatitis B virus (HBV). If these types of vaccines were possible, the onset caused by driver mutations could be delayed in healthy life. Validation of preclinical studies in spontaneous mouse models is required for protective vaccine usage. A recent study by Lu et al. suggested a novel approach to increasing responsiveness to immune checkpoint blockade (ICB) therapy. They provide preclinical evidence that pharmacological modulation of the spliceosome results in the generation of a substantial amount of highly immunogenic, splicing-derived neoantigens, augmenting the immune response in mice following ICB treatment [118].

For therapeutic clinical applications, an effective vaccination strategy and TCR-T transfer are expected. A number of vaccination strategies using neoantigen have not been thoroughly investigated. Optimal regimens for each patient will be required [119,120]. Well-designed vaccines against tumor neoantigens and tumor antigens, such as lipoprotein-mimicking nanodiscs, modified liposomes, and albumin-binding nanovaccines, should be applied to mouse preclinical models [121,122,123]. In particular, considering the characteristics of neoantigens that are recognized by both CD4^+^ and CD8^+^ T cells and their loss by HLA defects or LOH, simultaneous evoking of innate immunity and total adoptive immunity is effective. Our proposed vaccine, NKT ligand-loaded CD1d^+^ cells carrying the tumor antigens that target dendritic cells and induces activation of innate immunity and antigen-specific CD4^+^ and CD8^+^ T cell responses, named artificial adjuvant vector cells (aAVC), should be efficacious [124,125]. Patients who benefited from poor responses due to HLA loss by vaccine treatments are typically eligible, since the aAVC system also activates NK and iNKT cells [126]. Not limited to single usage of neoantigen vaccines, combination with radiotherapy enhances the clinical effects by modifying tumor gene expression and host immune responses [127].

A previous study showed that neoantigen-specific T cell (HLA-C*08:02 restricted KRAS G12D) transfer resulted in tumor rejection, but yielded tumor evasion due to loss of MHC-I in one lesion [96]. Contrary to expanded CTLs, TCR transgenic T cells targeting HLA-A*02:01 restricted NY-ESO1 have already shown drastic clinical responses in patients with refractory metastatic melanoma, synovial cell sarcoma, and multiple myeloma [128,129]. TCR transgenic CD4^+^ T cells targeting HLA-DPB1*04:01 restricted melanoma-associated antigen A3 (MAGE-A3) also showed efficiency in various cancer types [130]. Hence, TCR-T therapy against neoantigens is desired with validated safety and clinical responses. In addition, more effective administration regimens of TCR-T have been proposed, such as using iPSC technology, combination with ICB, pretreatment with chemical compounds, and CRISPR-Cas9-mediated genome engineering [131,132,133,134]. After establishing the confirmed TCR library against neoantigens, the replenished combination of neoantigen-specific TCR-T cells should provide an unprecedented complete cure for many human patients.

In principle, all cell surface molecules can be recognized by antibodies. Previous reports have shown that antibodies also act as TCR mimics that react with MHC-I/peptide complexes [135,136]. Recent technology has facilitated the identification of such MHC-I/peptide-specific single-chain variable fragments (scFv) [137,138,139]. In addition to antibody-dependent cellular cytotoxicity, reactive scFv sequences can be applied to chimeric antigen receptor (CAR)-T cells and bispecific antibodies with anti-CD3 antibody [140,141]. Beyond TCRs, tumor-specific neoantigens are the exact landmarks for selective tumor eradication.

In summary, for prospective neoantigen-based diagnosis and therapeutic use, much remains to be solved and optimized. Moreover, deep learning is important for the clear prediction of neoepitopes. For developing algorithms, more data from humans, especially regarding rare MHC alleles for versatile usage, and mice are needed. In addition, conceptual learning and deep learning may accomplish neoantigen prediction based on histology data, because computational image recognition is a strong tool of deep learning. In fact, deep learning would be able to be recognize MSI just based on images [142]. Given the spatially heterogeneous character of tumors, the combination of section image and NGS sequencing, including TCR analysis, will bring considerable progress. Collaboration of immunobiologists, medical professionals, informaticians, and AI engineers will lead to effective tumor therapy using neoantigens.

## Figures and Tables

**Table 1:** Clinical trials of neoantigens vaccines.

Clinical Trials	Tumor	Vaccines	Reference
NCT00683670	Melanoma	Dendritic Cell	Carreno et al., 2015 [60]
NCT01970358	Melanoma	Peptide	Ott et al., 2017 [61]
NCT02035956	Melanoma	mRNA	Sahin et al., 2017 [62]
NCT02149225	Glioblastoma	Peptide	Hilf et al., 2019 [63]
NCT02287428	Glioblastoma	Peptide	Keskin et al., 2019 [64]
NCT02510950	Glioblastoma	Peptide	Johanns et al., 2019 [65]
NCT02897765	Advanced melanoma/Non-small cell lung cancer/Bladder cancer	Peptide + aPD-1	Ott et al., 2020 [66]
NCT03171220	Thymoma/Pancreatic cancer	Dendritic Cell	Chen et al., 2019 [67]
NCT01461148	Colorectal cancer	Peptide	Kloor et al., 2020 [68]
NCT03480152	Gastrointestinal cancer	mRNA	Cafri et al., 2020 [69]
NCT03662815	Melanoma/Colon Cancer/Non-small cell lung cancer/Pancreatic Cancer/Biliary Tract Cancer/Ovarian Cancer	Peptide	Fang et al., 2020 [70]
NCT01970358	Melanoma	Peptide	Hu et al., 2021 [71]
NCT03645148	Advanced pancreatic cancer	Peptide	Chen et al., 2021 [72]
NCT02454634	Gliomas	Peptide	Platten et al., 2021 [73]

## Data Availability

Not applicable
